# SLEEP QUALITY AND ITS ASSOCIATION WITH PSYCHOLOGICAL SYMPTOMS IN ADOLESCENT ATHLETES

**DOI:** 10.1590/1984-0462/;2017;35;3;00009

**Published:** 2017-07-31

**Authors:** Gabriel Cordeiro Gomes, Muana Hiandra Pereira dos Passos, Hítalo Andrade Silva, Valéria Mayaly Alves de Oliveira, Wbinayara Alves Novaes, Ana Carolina Rodarti Pitangui, Rodrigo Cappato de Araújo

**Affiliations:** a Universidade de Pernambuco (UPE), Petrolina, PE, Brasil.; b UPE, Recife, PE, Brasil.

**Keywords:** Sleep wake disorders, Affective symptoms, Adolescent, Sport psychology

## Abstract

**Objective:**

To verify the prevalence of poor sleep quality and its association with personal characteristics and symptoms of depression, anxiety and stress in amateur adolescent athletes.

**Methods:**

309 adolescent athletes aged between 10 and 19 years were enrolled. Data collection included: a structured questionnaire, with personal information; the Pittsburgh Sleep Quality Index (PSQI); and the Depression, Anxiety and Stress Scale (DASS-21). Results are described in mean and standard deviation (numeric variables) and absolute and relative frequencies (categorical variables). For the inferential analysis, Student’s t-test and chi-square test were performed, in addition to Poisson regression. Prevalence ratios (PR) were calculated in a 95% confidence interval (95%CI).

**Results:**

The mean age of participants was 14.1±2.1, being 13.8±2.0 and 15.0±2.1, respectively, for those with good and poor sleep quality. Poor sleep quality was recorded in 28.2% (n=87), depression in 26.9% (n=83) and anxiety/stress in 40.1% (n=124). Poor sleep quality was associated with ages between 15 and 19 years (PR 1.24; 95%CI 1.14-1.37), overweight (PR 1.12; 95%CI 1.01-1.24) and psychological symptoms of depression (PR 1.23; 95%CI 1.08-1.40) and anxiety/stress (PR 1.16; 95%CI 1.04-1.28).

**Conclusions:**

The presence of overweight and psychological symptoms and the age over 15 years were risk factors for increasing the likelihood of poor sleep quality in adolescent athletes.

## RESUMO

**Objetivo:**

Verificar a prevalência de má qualidade de sono e sua associação com características pessoais e sintomas de depressão, ansiedade e estresse em adolescentes atletas amadores.

**Métodos:**

Foram avaliados 309 adolescentes atletas, entre 10 e 19 anos. Para a coleta de dados foram utilizados: questionário estruturado, contendo informações pessoais; Índice de Qualidade do Sono de Pittsburgh (PSQI); e Escala de Ansiedade, Depressão e Stress de 21 itens (EADS-21). Na análise descritiva foram calculados a média e o desvio padrão das variáveis numéricas e as frequências absolutas e relativas das variáveis categóricas. Para a análise inferencial foram realizados teste *t* de Student e teste do qui-quadrado, além de regressão de Poisson, sendo calculadas as razões de prevalência (RP) em um intervalo de confiança de 95% (IC95%).

**Resultados:**

A média de idade dos participantes foi de 14,1±2,1, sendo 13,8±2,0 para o grupo de adolescentes com boa qualidade do sono e 15,0±2,1 para o grupo com má qualidade do sono. A má qualidade do sono foi registrada em 28,2% (n=87), a depressão, em 26,9% (n=83) e a ansiedade/estresse, em 40,1% (n=124) da amostra. A má qualidade do sono se associou à faixa etária de 15 a 19 anos (RP 1,24; IC95% 1,14-1,37), a adolescentes com sobrepeso (RP 1,12; IC95% 1,01-1,24) e com sintomas de depressão (RP 1,23; IC95% 1,08-1,40) e de ansiedade/estresse (RP 1,16; IC95% 1,04-1,28).

**Conclusões:**

A presença de sobrepeso e sintomas psicológicos, bem como a idade superior a 15 anos, se mostraram fatores de risco para aumentar a chance da má qualidade do sono em adolescentes atletas.

## INTRODUCTION

Sleep is a physiological condition characterized by a reversible behavioral state, with changes in the level of consciousness and responsiveness to stimuli.^1^ In this context, studies have assessed the relationship between sleep and physiological mechanisms essential to life, such as the production of energy, neural plasticity and secretion of the growth hormone.^2^ ^,^ ^3^ In adolescence, sleep quality goes through significant changes due to different sources of influence, leading to insomnia, excessive daytime sleepiness, changes in sleep-wake cycle, and other disorders.^4^ ^,^ ^5^

Even though the need for sleep is an individual characteristic, an average of at least 8.3 hours of sleep per night is recommended for adolescents, to prevent excessive daytime sleepiness.^6^ Good sleep quality is essential for young athletes for ensuring better performance in psychomotor and cognitive activities, and for reducing the chances of developing risk factors for musculoskeletal pain.^7^ ^,^ ^8^ Nationally, it has been pointed out that 48.5% of young athletes sleep less than 8 hours a day, with prevalence of low sleep quality of 41.7%.^9^

Sleep loss during adolescence is not triggered by less need for sleep, but by a convergence of biological, psychological and sociocultural influences related to changes in circadian rhythm, autonomy to choose sleeping hours, academic pressure, use of screen devices and social network.^4^ It is worth to mention that the repercussions of poor sleep quality may be related with the reciprocity of psychological symptoms, such as depression, which can trigger suicidal thoughts among adolescents.^10^ Around the age of 15, about half of the psychiatric disorders can appear, indicating that this phase is marked by major changes, able to expose adolescents to situations of emotional vulnerability.^11^

Considering the need of good sleep quality for the neuropsychomotor development of adolescents and their cognitive and mental well-being, it is extremely important to study the association between poor sleep quality and the presence of symptoms of anxiety, depression and stress, in order to provide subsidies to health professionals who follow-up athlete adolescents in terms of physical and behavioral domains. Despite the existence of studies that show the association of poor sleep quality and psychological symptoms, there are still a few assessing the athlete adolescent population specifically. Therefore, this study aimed at verifying the prevalence of poor sleep quality and symptoms of depression, anxiety and stress among amateur adolescent athletes, besides assessing the association between poor sleep quality and personal characteristics and psychological symptoms.

## METHOD

This is an observational, correlational, cross-sectional study. The study included adolescents of both sexes, aged between 10 and 19 years, who were amateur athletes of different sports, with at least one year of practice, in the city of Petrolina, Pernambuco. Adolescents were considered amateur athletes when practicing their sports without earning any payment and who participated in regular or punctual competitions, in the educational level, promoted by municipal or state institutions. Adolescents who did not fill out the questionnaires adequately or refused to take the anthropometric measurements were excluded from the sample.

The number of amateur adolescent athletes was observed in 20 sports centers and schools of the city, reaching the total number of 521 individuals. Based on this population, sample size was calculated using the WinPepi software, version 11,6, with the following criteria: estimated population of 521 athletes, estimated prevalence of poor sleep quality of 37.2%, 10% sample loss, and design effect of 1.3; reaching a minimum sample of 308 participants.

The sampling procedure followed the following steps: the first stage was the observation of the total number of school and training centers that presented sports teams. The second stage included the clustered sampling procedure of sports modalities as sample units, considering the proportion of the modalities. The choice of the teams was defined by a list generated randomly in the WinPepi software, version 11,6, determining the sequence of schools or training centers that should be included until the minimum of subjects per modality was reached.

Finally, the data were collected in 10 institutions, including schools and training centers, reaching a final sample of 317 adolescents. The study included all eligible participants whose Informed Consent Form was signed by their tutors or participants older than 18 years old. The study was approved by the Research Ethics Committee at Universidade de Pernambuco, protocol CAAE 38321114.0.0.0000.520.

A structured questionnaire was elaborated to collect personal information, such as age and sex. Anthropometric measures of weight and height were collected and, right after that, the body mass index (BMI) of the individuals was calculated. The nutritional status of participants was categorized according to the criteria suggested by Cole et al.^12^ ^,^ ^13^

The Brazilian version of the Pittsburgh Sleep Quality Index (PSQI) was used to assess the sleep quality among athletes. The index was validated by Bertolazi et al.,^14^ who proposed to assess the quality of sleep of an individual for the period of one month. This questionnaire was validated for the adolescent population by Passos et al.^15^, and became a highly consistente internal tool - Cronbach’s alpha of 0.82 -, with moderate reliability in the evaluation of sleep disorders affecting this population. It is constituted of 7 components, which may be scored from 0 to 3, with a total score ranging from 0 to 21 points. Values higher than 5 indicate poor sleep quality.

The 21-item Depression, Anxiety and Stress Scale (DASS-21) was used to assess the presence of symptoms of depression, anxiety and stress among the participnats. The original version of this scale was translated and validated by Vignola and Tucci,^16^ which contains three domains to assess the emotional states of depression, anxiety and stress in the former week. This study used the version in Portuguese validated for adolescents,^17^ in which the constructs of anxiety and stress were assessed together, based on an exploratory factorial analysis of the scale items. It was observed that, in the adolescent population, the domains anxiety and stress presented items with strong factorial loads, making it difficult to discriminate both, and allowing better data analysis with only two domains: depression; anxiety and stress. Cronbach’s alpha coefficient for the domains of anxiety and stress were 0.82, and for depression, 0.80. Besides, the results of the instrument were reorganized in a dichotomous variable, defined by either the presence or the absence of symptoms.

The data were inserted in the software Microsoft Excel, and the analysis was conducted using the Statistical Package for the Social Scienes (SPSS), version 2.0. The Kolmogorov-Smirnov test verified the normality of data. In the descriptive stage of the analysis, mean and standard deviation of numerical variables were calculated, as well as the absolute and relative frequencies of categorical variables. The analytical stage included the Student’s *t* Test for independent samples, in order to assess the existence of significant differences between the numerical variables in both groups of adolescents, with good and poor sleep quality. Besides, the chi-square test was conducted to verify the difference between the frequencies of categorical variables in both groups, and also to examine the association between both variables. The Poisson Regression was also used, with robust variance, to calculate the prevalence ratio (PR) and its respective 95% confidence intervals (95%CI). The Omnibus test was also carried out to verify the significance of the developed models. The statistical significance level was considered in 5% for the analyses.

## RESULTS

We collected data from 317 amateur adolescent athletes; however, 8 participants were excluded for not filling out the instruments completely, leading to a final sample of 309 adolescents. The data describing this sample, stratified as to the classification of sleep quality, are presented in Table 1.

**Table 1:**
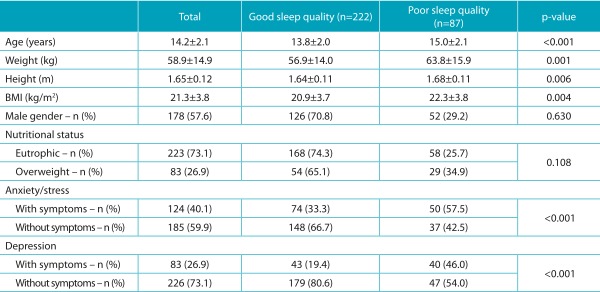
General characteristics of the total sample and groups of adolescentes with good and poor sleep quality.

BMI: body mass index.

Regarding the sports modalities, 31.7% (n=98) practiced volleyball; 25.2% (n=78), handball; 18.4% (n=57), basketball; 13.6% (n=42), swimming; and 11% (n=34), judo. Poor sleep quality affected 87 (28.2%) adolescents; psychological symptoms of depression were observed in 83 (26.9%); and anxiety/stress symptoms, in 124 (40.1%).

Table 2 presents the results in the Poisson regression model, with the PR values of the variables sex, age, BMI and symptoms of depression and anxiety/stress regarding poor sleep quality. This model presented satisfactory results in the Omnibus test (*p*<0,001).

**Table 2:**
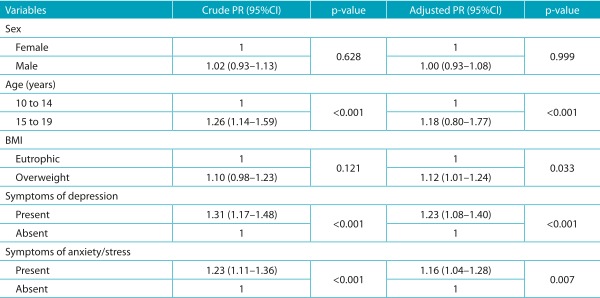
Crude and adjusted prevalence ratio for factors associated with poor sleep quality in amateur adolescent athletes.

PR: prevalence ratio; 95%CI: 95% confidence interval; BMI: body mass index.

## DISCUSSION

The health of adolescents has been analyzed more and more often, and their sleep patterns are a matter of concern. In this study, the prevalence of poor sleep quality was 28.2%. This prevalence is different from the result found by Paiva et al.,^18^ in which poor sleep quality was registered in 37.2% of the adolescents assessed. It is important to mention that such divergence can be explained by the different populations evaluated, since this study analyzed amateur adolescent athletes, and physical exercise, when practiced regularly, promotes benefits for sleep quality.^18^ An investigation conducted with young athletes showed that the prevalence of poor sleep quality was 62.3%; however, participants were assessed prior to a competition. It is known that excitement and anxiety favor the occurrence of poor sleep quality, prolonged sleep latency and night waking in the nights preceding a sports evaluation or competition.^18^

Among the psychological symptoms assessed in the present sample, there was a prevalence of 26.9% for depression and 40.1% for anxiety/stress. The high prevalence of psychological disorders is in accordance with epidemiological studies that show an increasing pattern in the prevalence of these symptoms among adolescents.^19^ Data collection regarding psychological symptoms using scales and questionnaires can be one of the explanations for this finding. Such methods facilitate the overestimation of mild and momentary mood disorders, in comparison to more conventional methods, such as interviews. However, the acquisition of these data from self-reports is indispensable, since it informs the unique perception of the individual about his or her own behavior.^20^

Complaints about sleep quality are more common among adolescents with depression, who require more attention for reporting suicidal and self-harm intentions.^21^ In this study, the athlete adolescents who presented with poor sleep quality had higher chances of reporting symptoms of depression. The explanation for this association may lie on the fact that lack of sleep leads to the inappropriate modulation of emotional cerebral responses to aversive stimulation, since young people who suffer from sleep deprivation show hyper-limbic response through the amygdala as a consequence of exposure to more and more negative imaging stimuli.^21^ It is worth to mention that the lack of sleep has been pointed out as a factor of interference in the ability of adolescents to deal with the daily stress, and as a harmful aspect of their relationships with colleagues and adults.^22^

The study by Jin et al.^23^ evaluated 5,259 Chinese adolescents and young adults aged between 13 and 26 years, and concluded that 14.1% of them reported symptoms of anxiety alone. In the present study, in a different manner, adolescents presented both anxiety and stress, and both were included in a single category (anxiety/stress). Athlete adolescents with poor sleep quality also have more chances of reporting symptoms of anxiety/stress. In a study conducted with high school students in periods of one month, one week and one night before sports competitions, poor sleep quality was observed, as well as prolonged sleep latency and more moments of night waking in the night prior to the evaluation, in relation to the prior week or month,^17^ which may have been caused by the stress to which adolescent athletes are subjected at a time of a competition.^24^

Among the personal variables associated with poor sleep quality, it was observed that older adolescent athletes and those overweight had higher chances of reporting poor sleep quality. By corroborating these findings, a meta-analysis that assessed the patterns of sleep on the health of adolescents showed that sex, age, and geographic region impact the sleep patterns. Therefore, girls tend to sleep 11 extra minutes per night in relation to boys; adolescents tend to lose 14 minutes during the day and 7 minutes at night in the duration of sleep each year; and Asian adolescents sleep approximately 40 to 60 minutes less than Americans, and 60 to 120 minutes less than Europeans.^25^

This study found more chances of older adolescents, aged between 15 and 19 years, reporting poor sleep quality, corroborating the study by Felden et al.,^26^ who identified that the risk of poor sleep quality increased with age. This association may be explained by the fact that, while there is a cumulative increase of academic and recreational activities, there is also a reduction of time of sleep throughout the years.^27^ Besides, the delay in the biological process of melatonin secretion, hormone that is responsible for controlling the sleep-wake cycle, is related with the advancement of puberty and with the reduced sleep hours among adolescents.^22^

About the higher probability of overweight adolescents reporting poor sleep quality, obesity is not only related with the lack of diet or physical inactivity, but also with the negative perception of sleep.^28^ Among them, sleep deprivation seems to be associated with increasing stimuli to activate the orbitofrontal cortex, insula, thalamus, precuneus, cingulate gyrus, supramarginal gyrus, which are related with motivation and the value of food reward, as well as cognitive processing, decision-making and self-control. Therefore, sleep deprivation would change the neuron activity, making individuals more prone to food stimuli, which partly explains the association between poor sleep quality and high BMI values.^29^ In addition, other mechanisms are described in the literature as being responsible for the association between lack of sleep and increasing weight, such as changes in appetite hormones. Besides, insufficient sleep tends to affect energy consumption and use, resulting in more time and opportunities for the intake of food, especially high calorie items.^30^

The limitations of this study are related with its cross-sectional design, which does not allow to establish a causality relationship between personal characteristics, psychological symptoms and poor sleep quality. Besides, the dichotomization of results in the EADS-21 scale and the sum of the domains of anxiety and stress may have overestimated the prevalence of psychological symptoms. It is important to mention that the evaluation of sleep quality among the participants did not consider the calendar of sports activities, therefore not distinguishing periods of training, pre-competition and competition, and it is a known fact that these moments may influence the athletes’ sleep quality.

Finally, by concluding that good sleep quality is essential for both physical and mental well-being, especially for athlete adolescents, it is suggested that further studies assess the efficacy of the insertion of techniques and methods aiming at relaxation and development of cognitive health in training routines. The objective would be to preserve the behavioral and psychological aspects of the adolescents. 

The findings in this study indicate that athlete adolescents present with high prevalence of poor sleep quality and symptoms of depression and anxiety/stress. Besides, there was significant association between poor sleep quality with age, BMI and the psychological symptoms assessed.
